# Supplementary material for: Antitumor Effects of Ral-GTPases Downregulation in Glioblastoma
Source: Int J Mol Sci. 2022 Jul 25;23(15):8199. doi: 10.3390/ijms23158199 (PMC9330696; doi:10.3390/ijms23158199)

Figure S1.

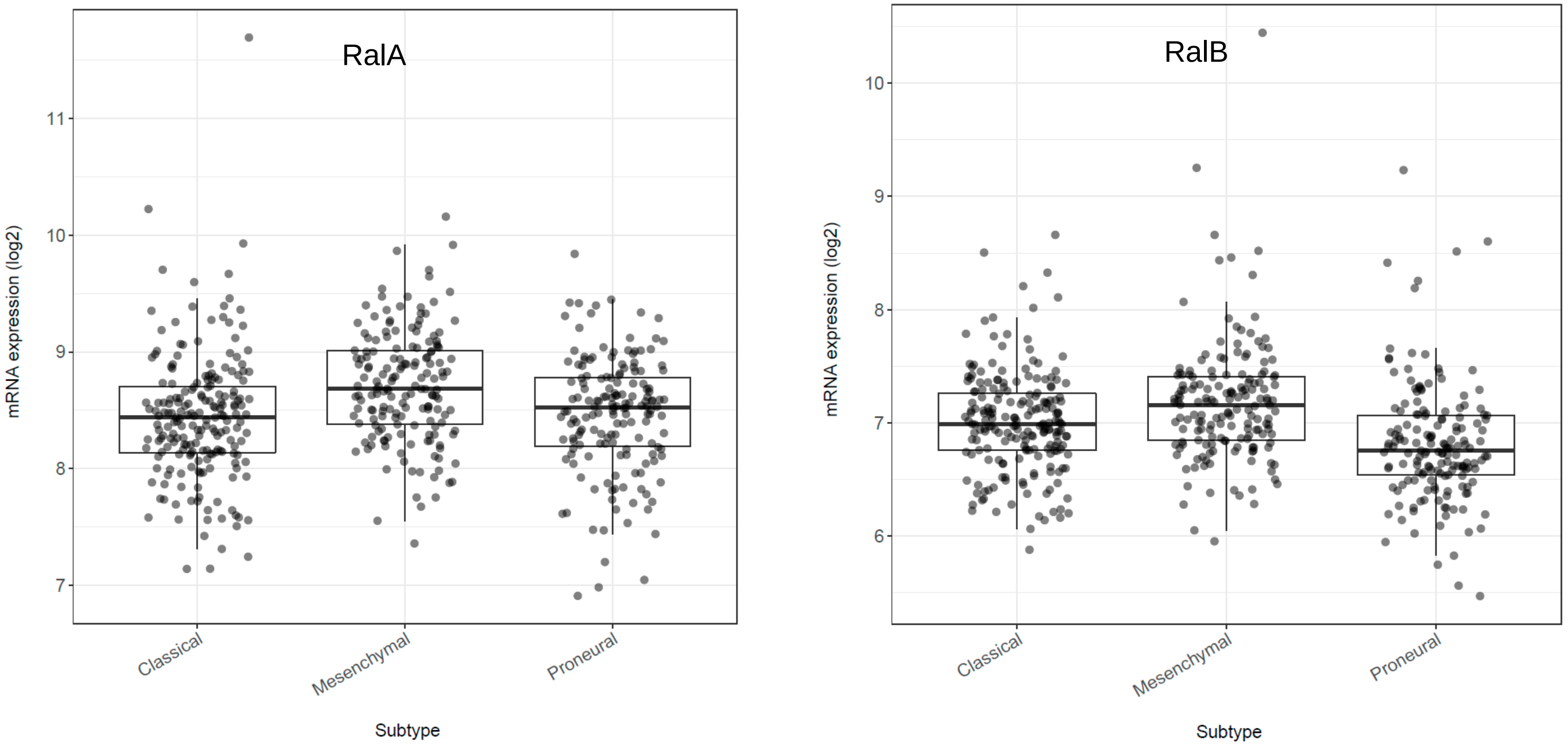

Figure S1 . *RalA and RalB expression in different GBM subtypes.* Analysis of RalA and RalB mRNA expression levels from GlioVis database comparing three GBM types, Mesenchymal, Classical and Proneural (TGCA-GBM; platform HG-U133A). Classical n=199; Mesenchymal n=165; Proneural n=162. (http://gliovis.bioinfo.cnio.es/).

Figure S2.

(a)

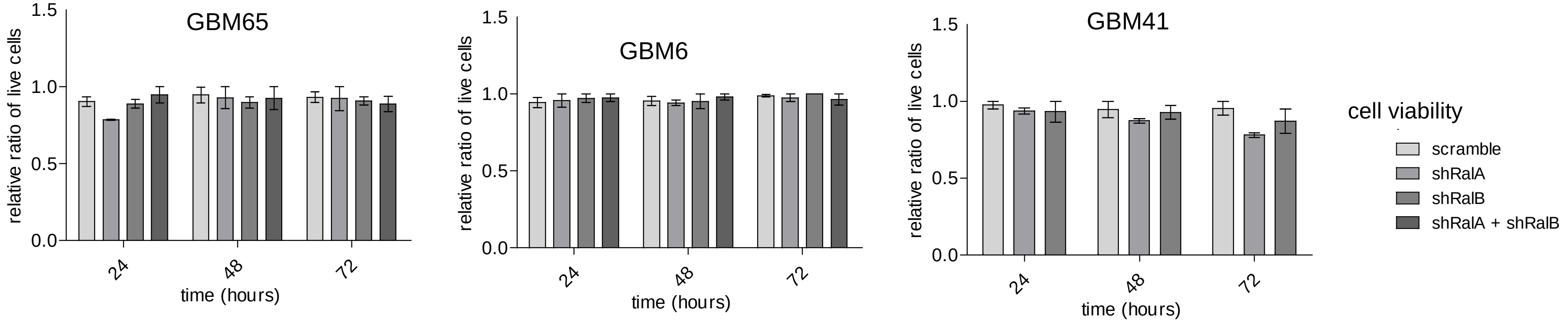

(b)

scramble shRalB

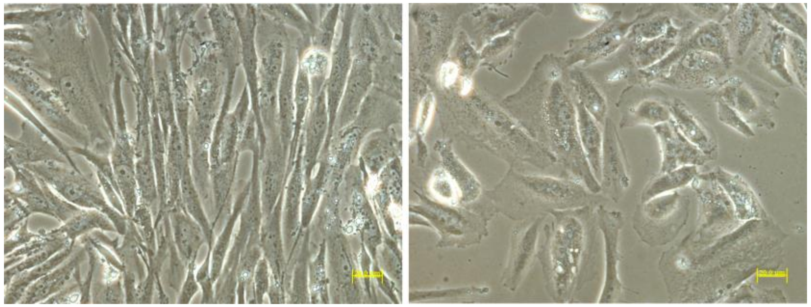

GBM6

(c)

scramble shRalB

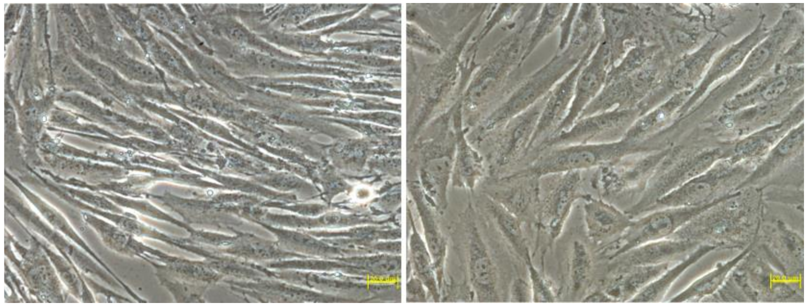

GBM41

Figure S2. *Knockdown of Ral GTPases promotes a reduction of cell growth without affecting cell viability*. Primary GBM cells were infected with lentivirus driving interference RNA against RalA (shRalA) or RalB (shRalB) or both. Scramble shRNA was used as a control. Three days after infection, cells were seeded and counted every 24 h for 3 days. **(a)** Graphics and bar diagrams representing trypan blue-negative GBM cells. Data is represented as mean ± SEM (n=3; three independent experiments). There was no significant differences. (**b, c)** Representative phase-contrast images of GBM6 (b) and GBM41 (c) cells were taken after 4 days of infection (20 μm Bar).

Figure S3

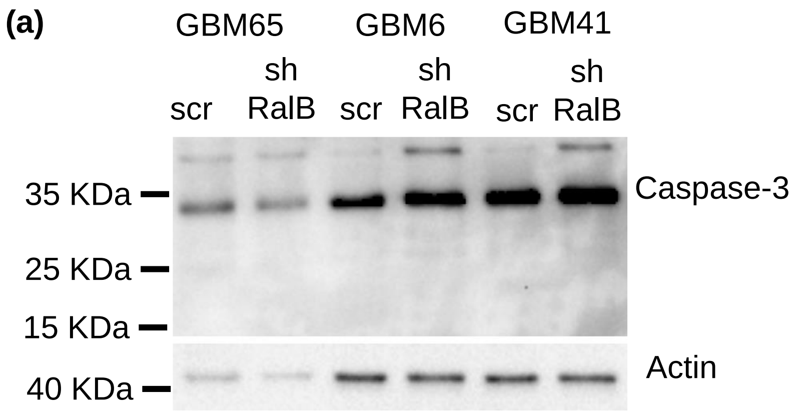

Figure S3. *Caspase-3 was not processed after downregulation of RalB in glioblastoma cells*. RalB was downregulated by RNA interference (shRalB). GBM cells were infected by lentiviral vectors harboring shRalB. Scramble shRNA was used as a control. Five days after infection cells were processed for immunoblot to detect total and cleaved caspase-3. Actin is used as a loading control

Figure S4

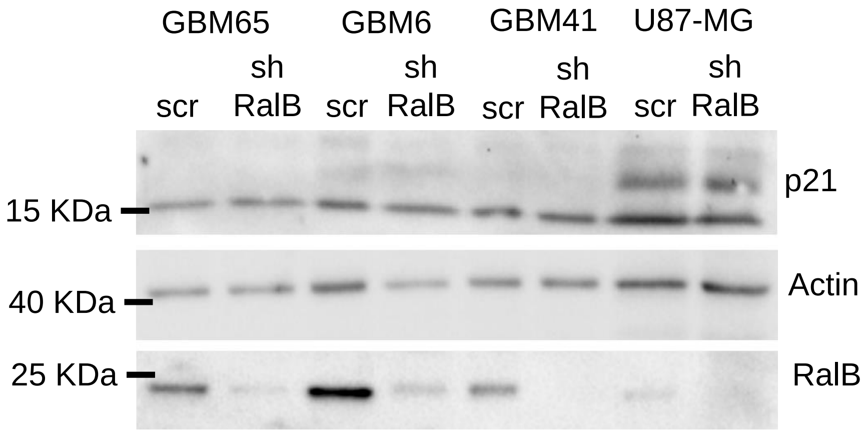

Figure S4. *p21 was not detected by immunoblot in the primary glioblastoma cells used in this work.* Primary GBM cells were infected with lentivirus driving interference RNA against RalB (shRalB). Five days after infection, cells were processed for immunoblot. Scramble shRNA was used as a control. Immunoblots of p21, actin (loading control) and RalB. The U87-MG GBM cell line was used as a positive control of p21 detection.

Figure S5

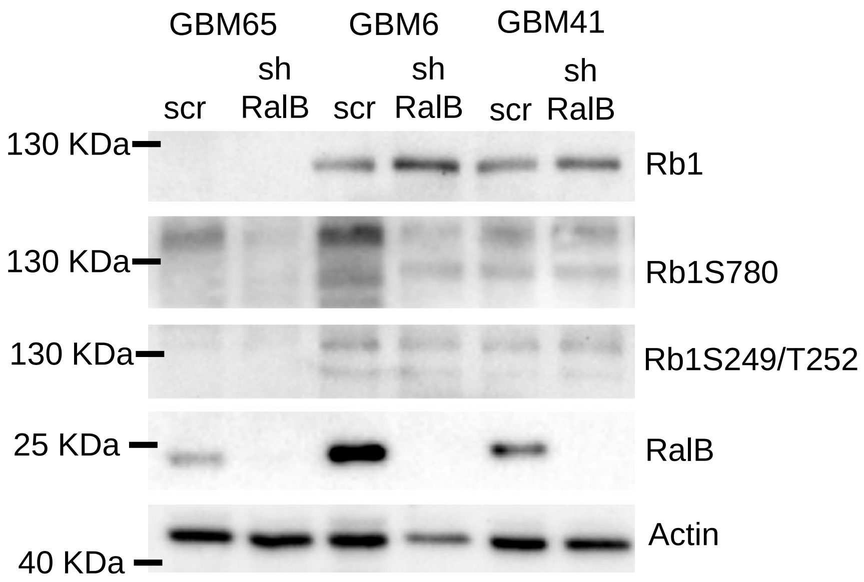

Figure S5. *Rb1 was hypophosphorylated in primary GBM cells after RalB-kockdown.* Primary GBM cells were infected with lentivirus driving interference RNA against RalB (shRalB). Five days after infection, cells were processed for immunoblot. Scramble shRNA was used as a control. Specific antibodies against Rb1, Rb1-phosphoS780, Rb1-phosphoS249/T252, actin (loading control) and RalB were used.

Figure S6

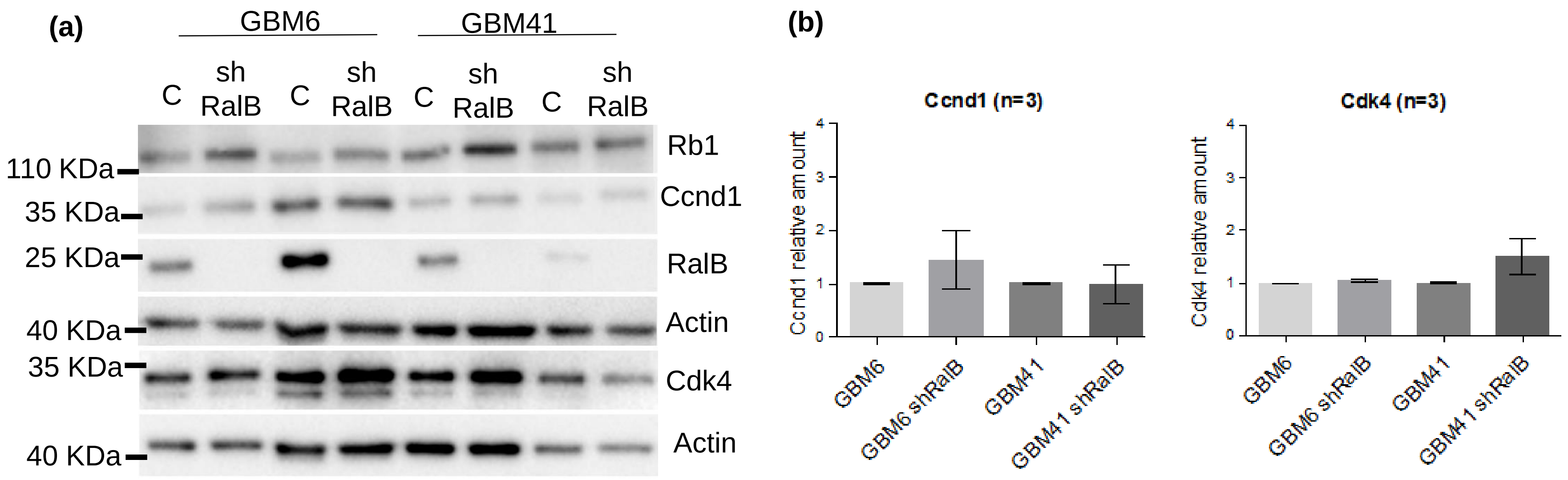

Figure S6. *Cyclin d1 and Cdk4 levels were not altered in primary GBM cells after RalB-knockdown.* Primary GBM cells were infected with lentivirus driving interference RNA against RalB (shRalB). Five days after infection, cells were processed for immunoblot. Scramble shRNA was used as a control. (a) Specific antibodies against Rb1, Ccnd1, Cdk4, actin (loading control) and RalB were used. (b) Bar diagrams representing the relative amount of Ccnd1 and Cdk4. Data is represented as mean ± SEM (n=3; three independent experiments) There was no significant differences.

Figure S7

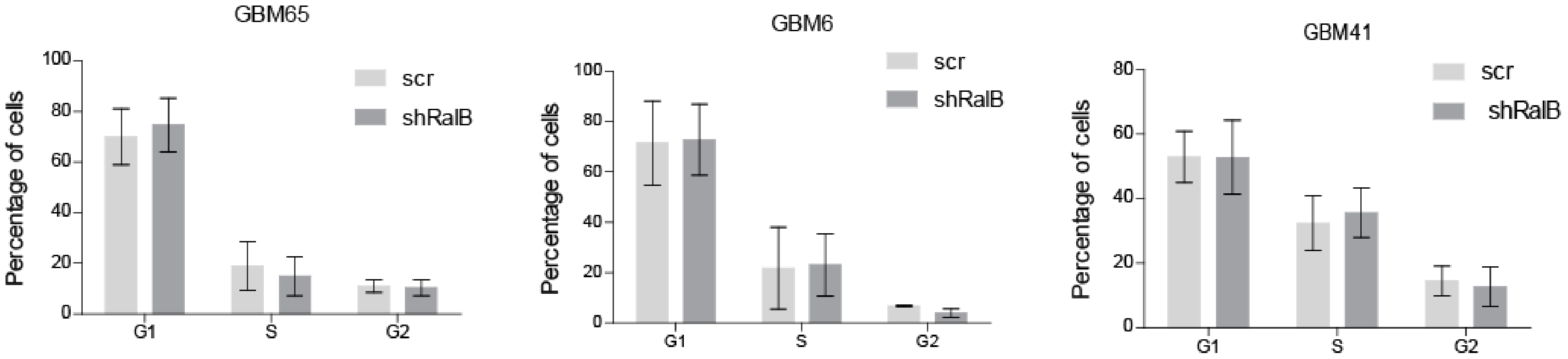

Figure S7. *RalB-knockdown did not produced a G1 arrest in primary GBM cells.* Primary GBM cells were infected with lentivirus driving interference RNA against RalB (shRalB). Five days after infection, cells were processed for FACS analyses. Scramble shRNA was used as a control. Bar diagrams representing the cell distribution in cell cycle phases examined by staining with PI. Data is represented as mean ± SEM (n=3; three independent experiments). There was no significant differences.

Figure S8

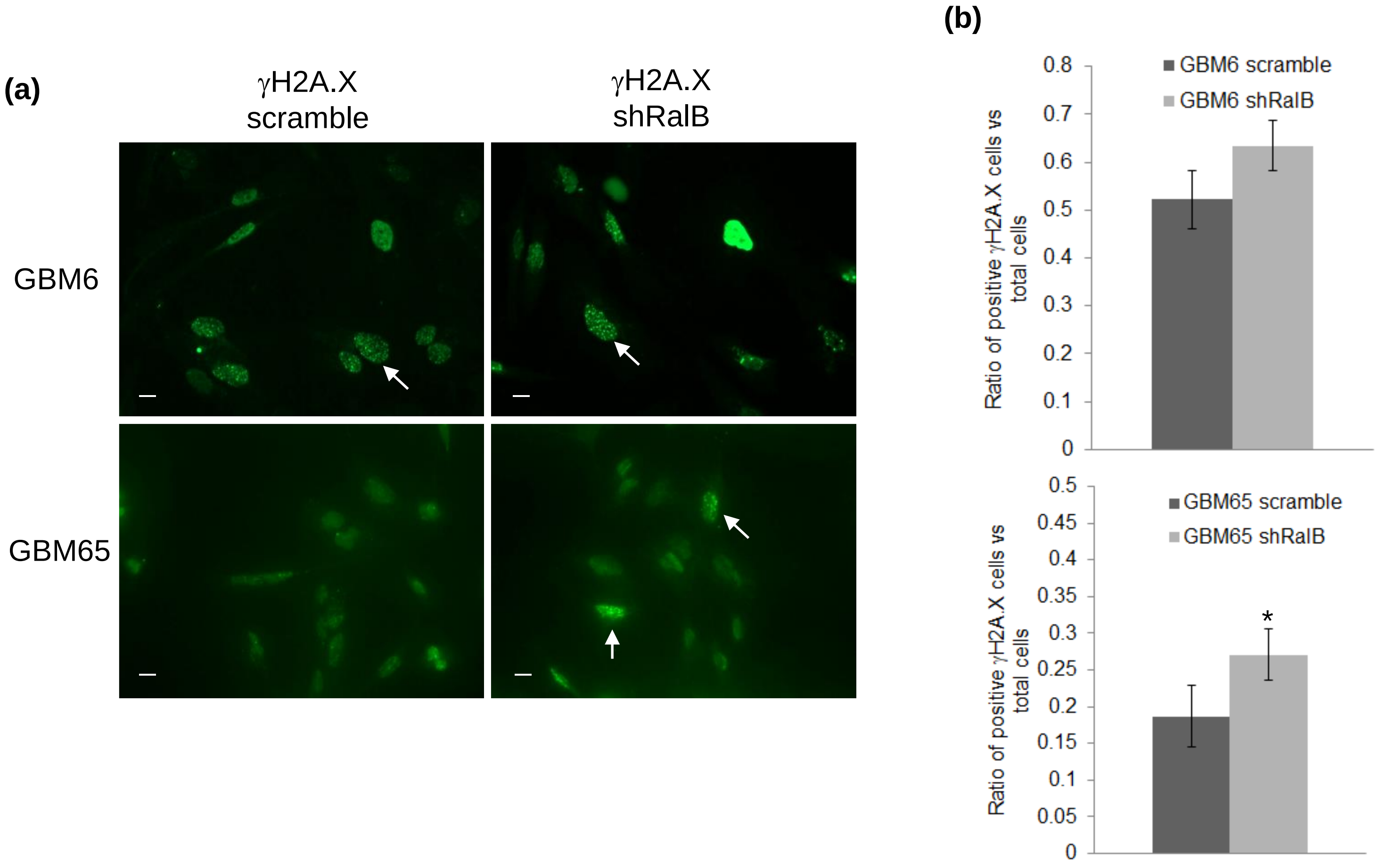

Figure S8. *DNA damage in glioblastoma cells after downregulation of RalB.* Primary GBM cells growing under the same conditions as in figure S4 were analyzed. (a) Representative images of cells positive for γ-H2A.X staining (20 μm Bar) (b) Cells with more than five foci were counted as positive. Graphs represent the ratio of positive cells versus total cells (n>255). The confidence intervals of proportions are shown (p≤0.05, *).

Figure S9. Uncropped blots of Figure 1a

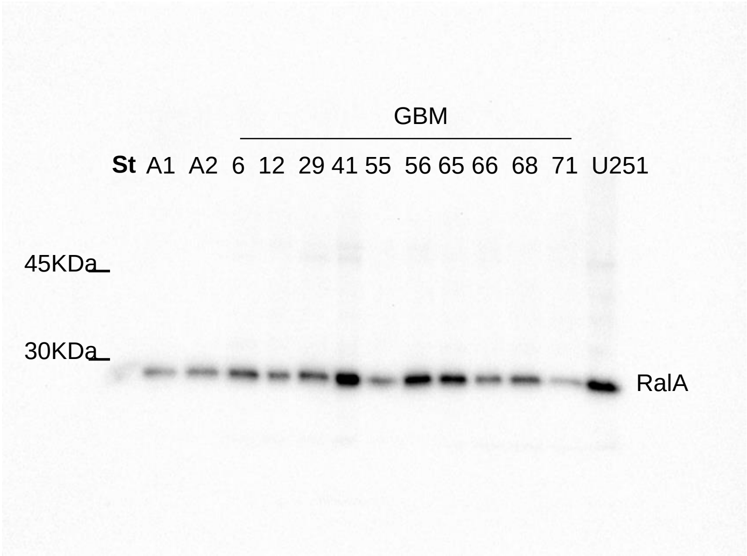

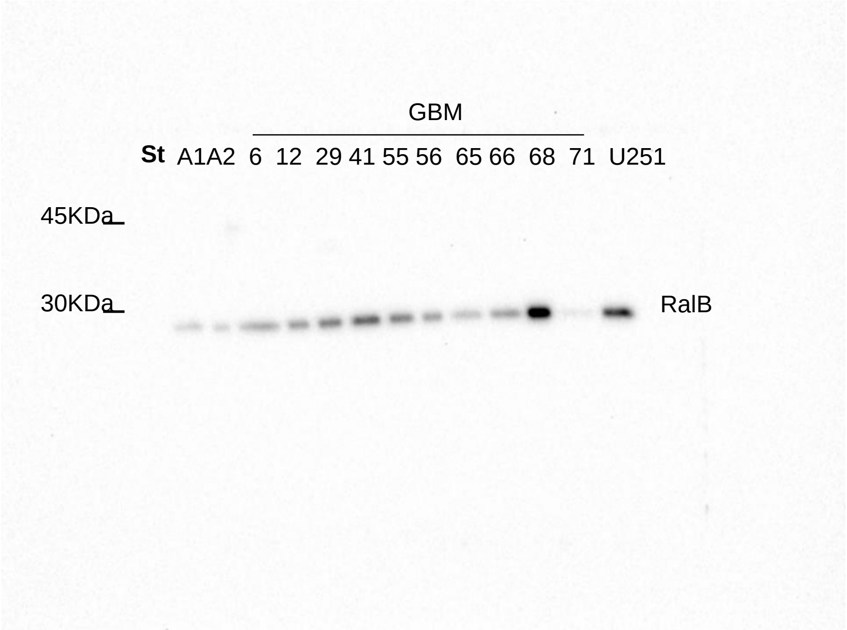

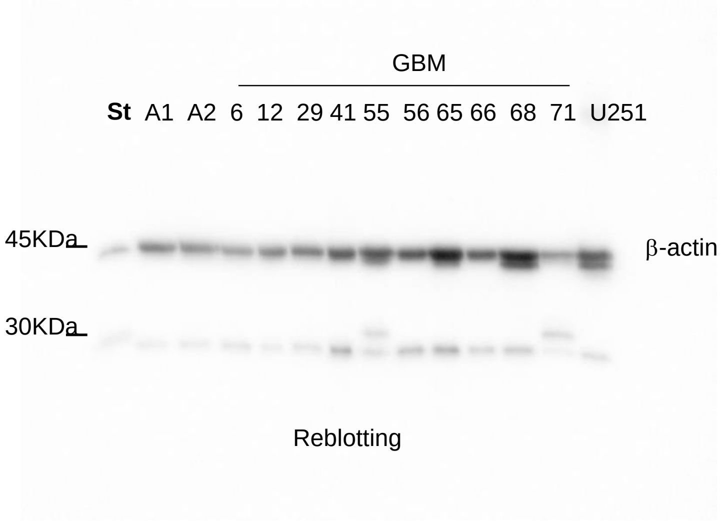

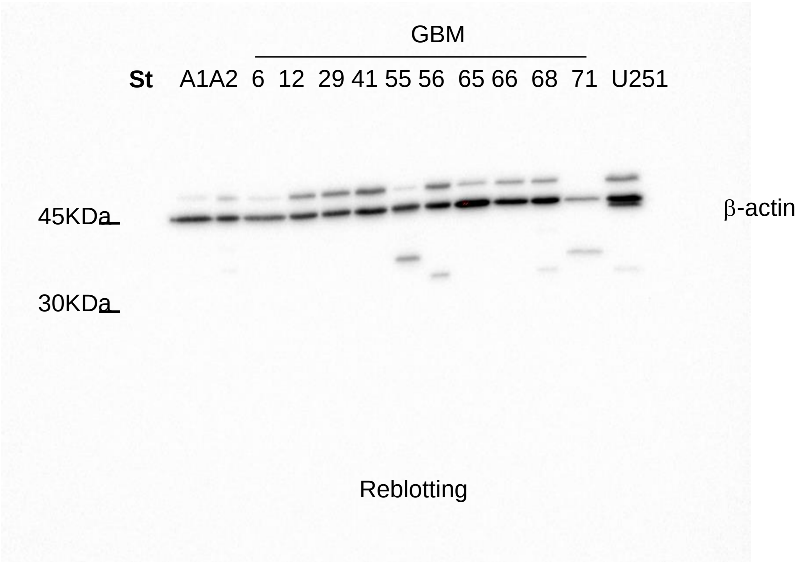

Figure S10. Uncropped blots of Figure 1b

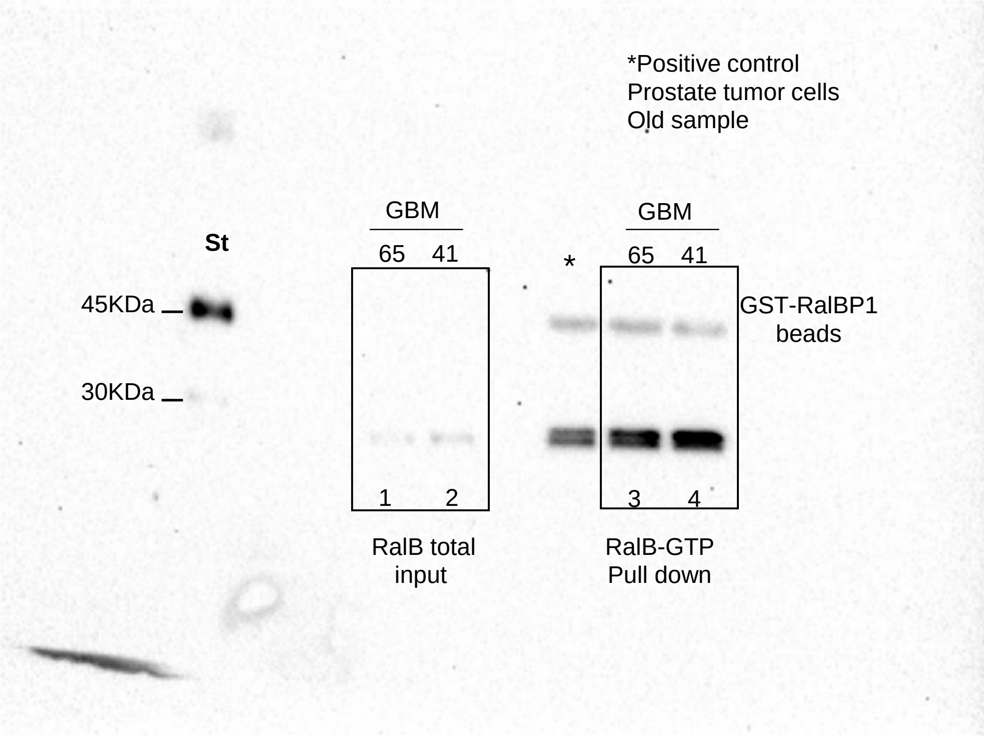

Figure S11. Uncropped blots of Figure 2b

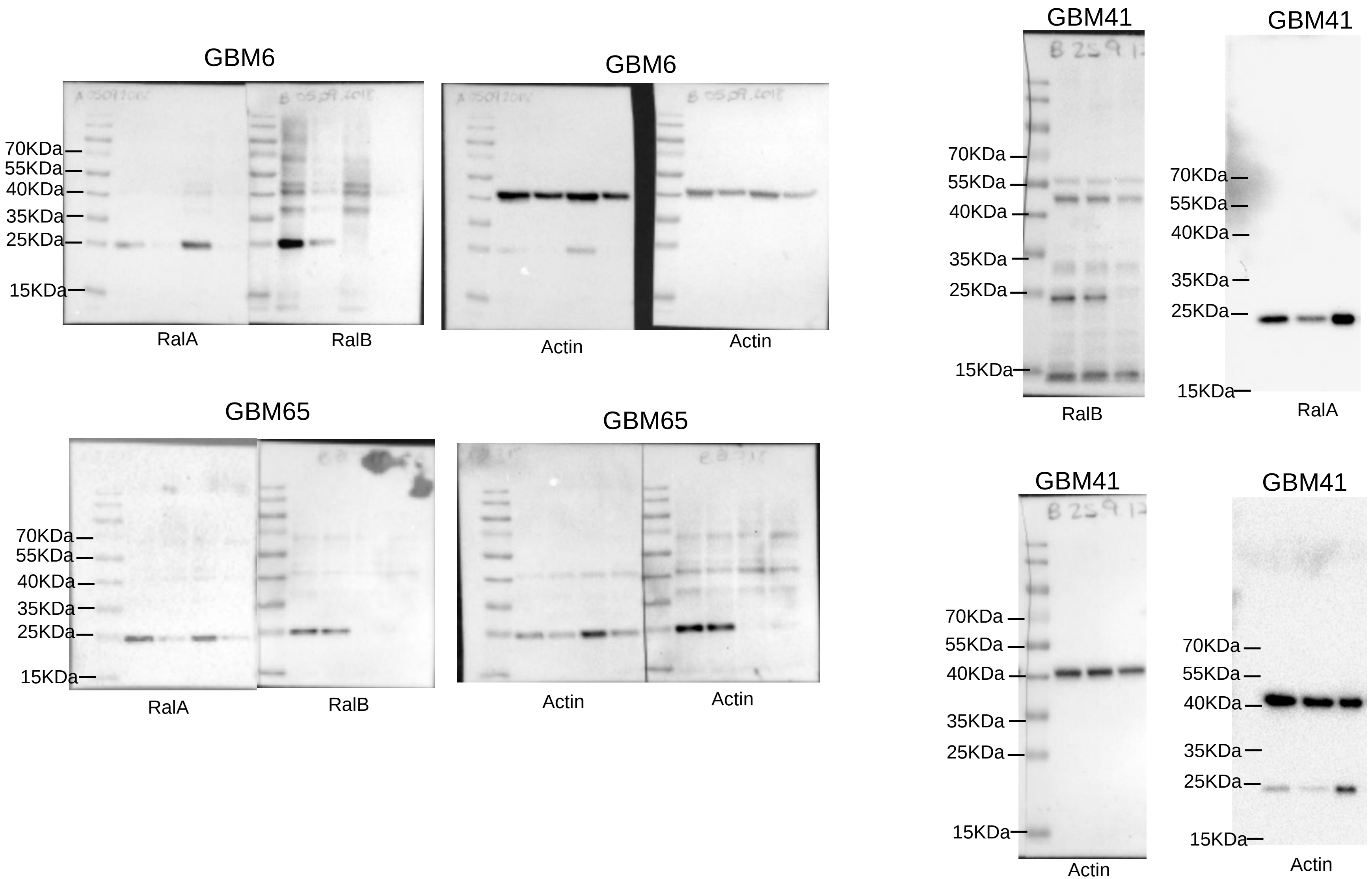

Figure S12. Uncropped blots of Figure 5a

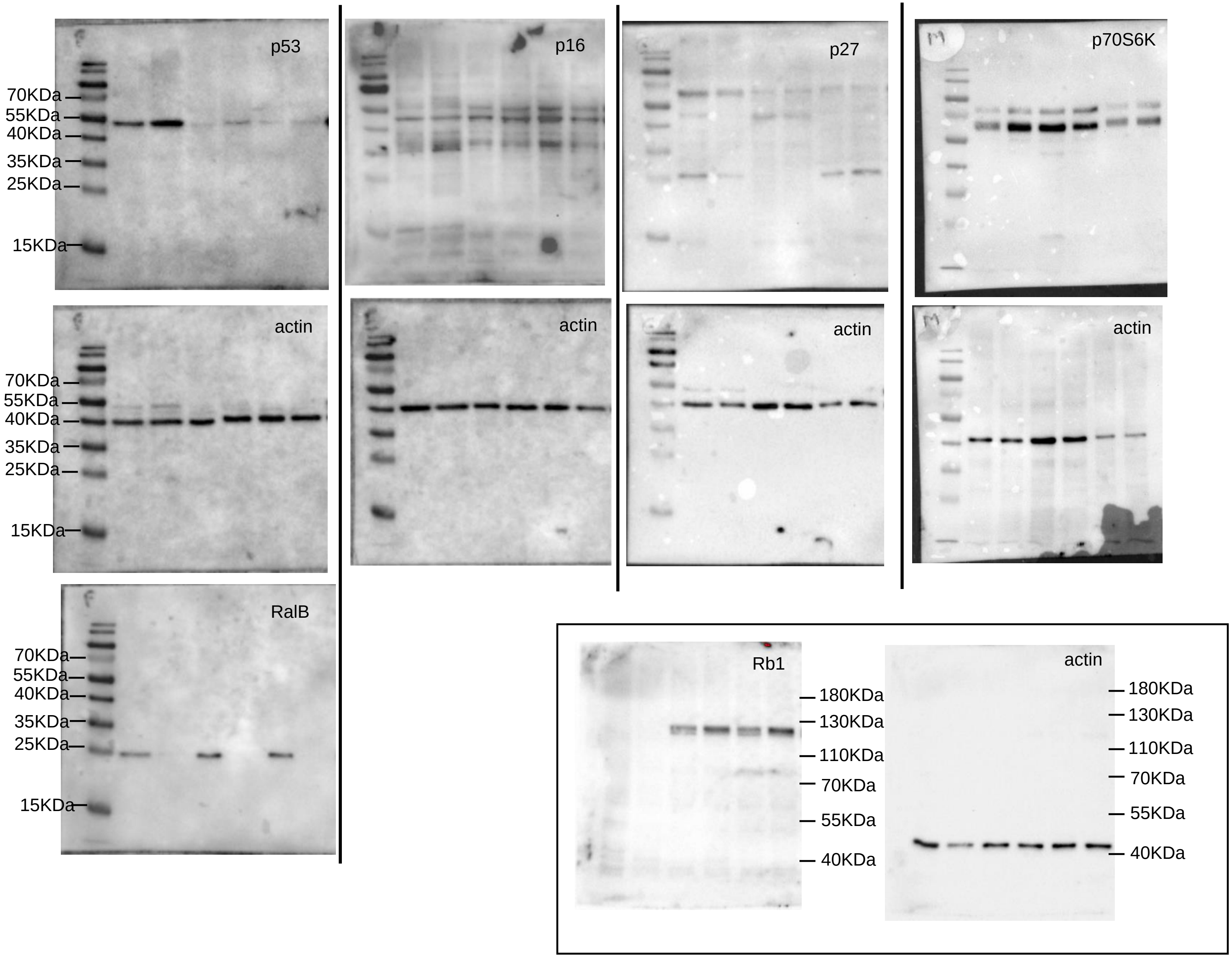

Figure S13. Uncropped blots of Figures S3 and S4

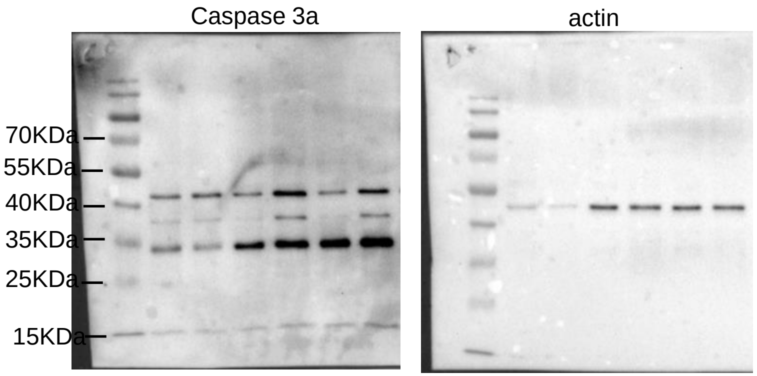

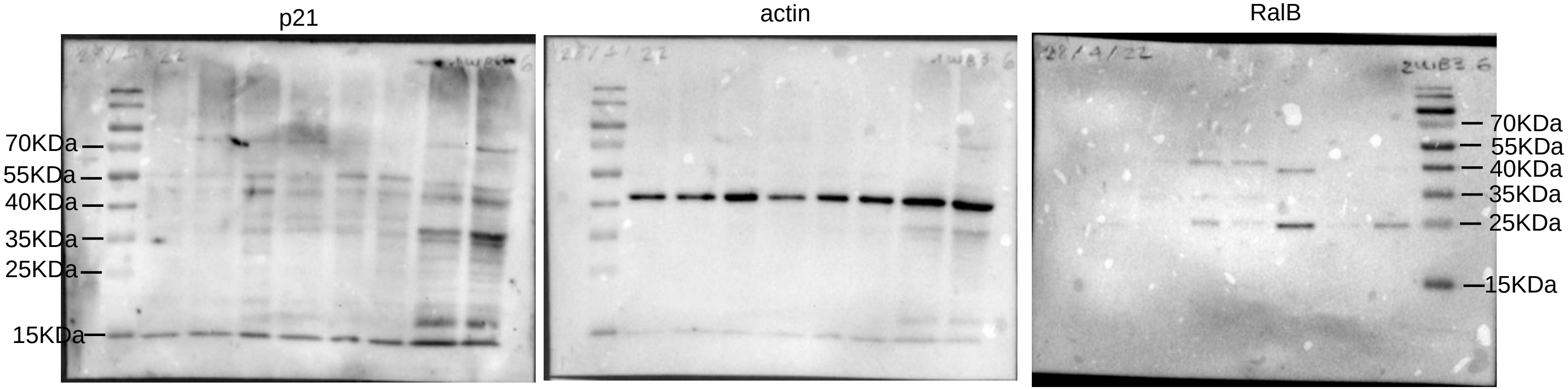

Figure S14. Uncropped blots of Figures S5and S6

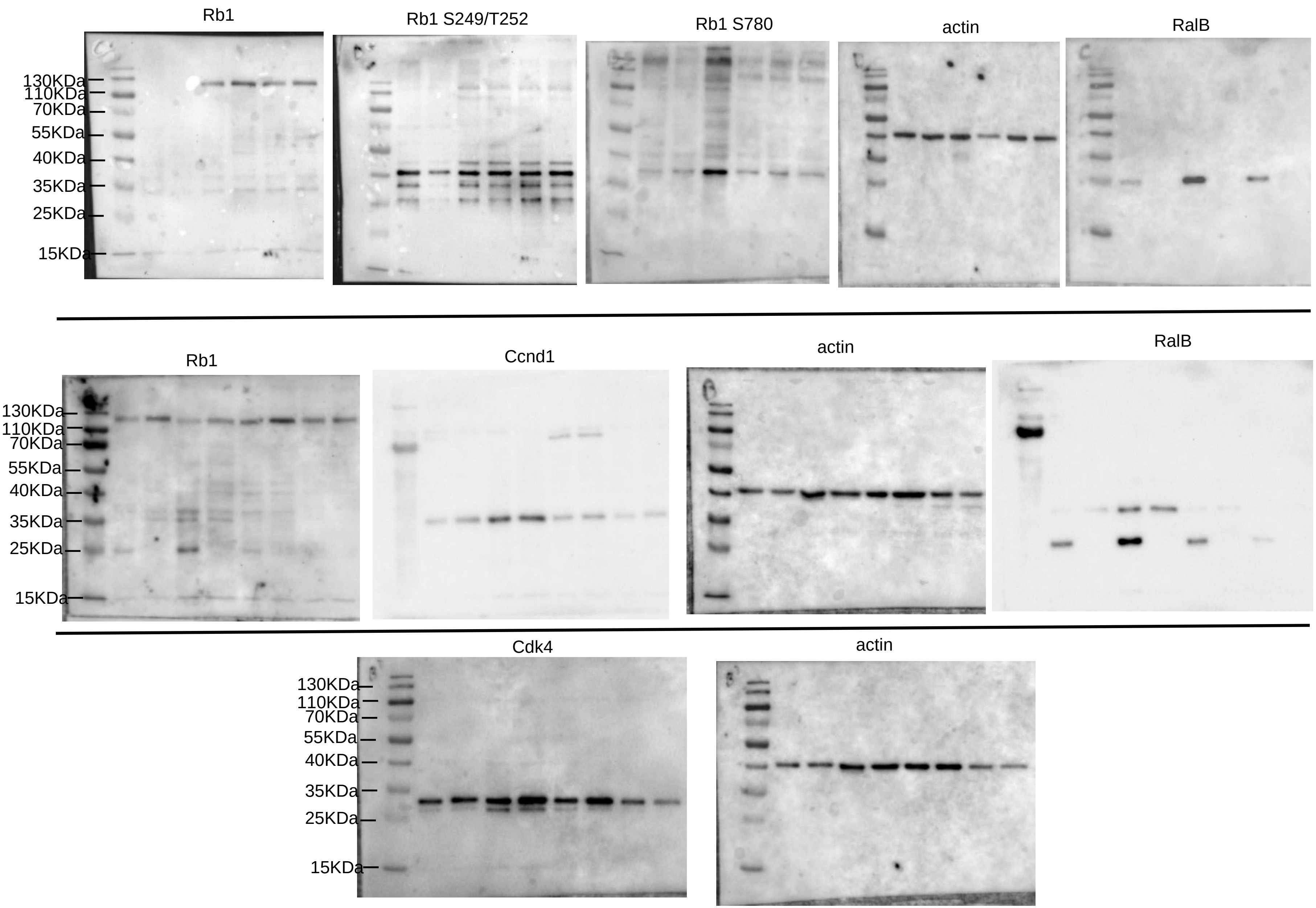

Supplement: Supplementary file 1 [file ijms-23-08199-s001.zip › ijms-1817779-supplementary.pdf]
